# Validation of the Korean Version of the COVID-19 Phobia Scale (K-C19PS)

**DOI:** 10.3390/ijerph18073747

**Published:** 2021-04-03

**Authors:** Mihyeon Seong, Misoon Lee, Insook Kim, Miran Kang

**Affiliations:** Department of Nursing, Changshin University, Changwon 51352, Korea; lms3021@hanmail.net (M.L.); dongyangmiin@cs.ac.kr (I.K.)

**Keywords:** COVID-19, Korea, phobia, reliability, scale, validity

## Abstract

The purpose of this study was to evaluate the reliability and validity of a Korean version of the 20-item COVID-19 phobia tool, which was developed through a translation-reverse translation process. These data were collected from 226 persons using a self-reported questionnaire. Exploratory and confirmatory factor analyses were used to test construct validity. Finally, for 19 out of 20 items, the item-level convergence and differential validity were confirmed. In addition, the reliability and validity of the tool as a whole has been verified. For the subscales, Cronbach’s α was 0.90 for psychological, 0.87 for psychosomatic, 0.86 for economic, and 0.87 for social. Appropriate reliability was confirmed. Correlations between the COVID-19 phobia tool and fear of COVID-19 confirmed validity. The Korean version of the COVID-19 phobia tool is an appropriate scale for measuring the fear of COVID-19 and relevant psychological characteristics. Therefore, future studies in areas such as health and nursing could use this tool as required.

## 1. Introduction

Since the first report of a coronavirus disease-2019 (COVID-19) caused by severe acute respiratory syndrome coronavirus 2 (SARS-CoV-2) in December 2019, the World Health Organization (WHO) declared the disease a global pandemic [1], and infections caused by the novel coronavirus are still an ongoing threat [2]. In South Korea, the first patient with COVID-19 was confirmed on 19 January 2020, and the transmissibility of the virus was markedly stronger than that of the Middle East respiratory syndrome coronavirus (MERS-CoV) that broke out in 2015 in South Korea [3,4].

This situation has had a grave psychological impact on all individuals worldwide [5], and most governments have announced the implementation of public policies regarding social distancing, quarantine, and self-quarantine [6]. The massive media coverage focused on the number of infections and deaths has taken a psychological toll on many people worldwide, such as by causing anxiety [5]. Due to the extremely high infection rate and relatively high mortality rate, people are anxious about the disease and are afraid of coming in contact with individuals who have potentially contracted COVID-19 [7]. According to a previous study, people are more concerned about COVID-19 than seasonal influenza [8], highlighting the importance of considering the psychological aspects of the pandemic in addition to the treatment and preventive measures for it [5].

Phobia refers to the continuous and excessive fear of an entity or situation [9]. Fear may amplify the damage inflicted by disease [10], and continuous fear may trigger a phobia [9]. However, the major focus of global societies regarding COVID-19 has been the development of vaccines, treatment, and infection control, with the psychosocial aspects being neglected [11]. Wang et al. [12] indicated that 16.5% of subjects reported depression, and 28.8% had anxiety symptoms, while Hao et al. [13] reported that people experience psychological distress in pandemic situations. In addition, several studies have reported that COVID-19 causes mental health problems, such as post-traumatic stress disorder and depression [14,15]. As the COVID-19 situation continues, people may feel anxious, and persistent and excessive fear of the disease can manifest as a phobia [9,12].

Since psychological aspects, such as fear and phobia, are important factors that must be considered in addition to COVID-19 treatment and infection control, there is a critical need to pay attention to these factors and conduct relevant research. Accordingly, Arpaci et al. [16] developed the COVID-19 Phobia Scale (C19P-S) to measure COVID-19 phobia. In addition, various tools have been developed to measure levels of anxiety, stress [17], and fear [10]. However, among these measurement tools, only C19P-S evaluates the fear response to COVID-19. C19P-S was developed according to the definition and diagnosis of phobia in the DSM-5, and the multidimensional structure of the tool is supported by studies of anxiety and fear [18].

Arpaci et al.’s [16] tool was determined to be reliable and valid in the United States. However, there is no corresponding scale to measure COVID-19 phobia in South Korea. In Korea, the only tool suitable to assess the psychosocial aspects of COVID-19 is that of Seong et al. [19]; this instrument is limited as it can only measure a single aspect of fear. Therefore, it is necessary to verify the validity and reliability of a Korean version of Arpaci et al.’s [16] tool, so it may be used to observe psychosocial aspects of Korean persons that currently cannot be assessed in a rigorous, valid manner. Thus, this study adapted the scale into the Korean language and validated its psychometric properties, so the scale may be utilized in clinical practice, thereby laying a foundation for evidence-based nursing care.

### Objective

The aim of this study was to adapt the C19P-S developed by Arpaci et al. [16] into Korean and validate the Korean version of the C19P-S (K-C19PS).

## 2. Method

### 2.1. Study Design

This study translated the C19P-S tool developed by Arpaci et al. [16] into Korean while considering the Korean social context and confirmed the validity and reliability of the Korean version.

### 2.2. Study Population

The study population for validating the K-C19PS was adults aged 20 years or older nationwide. Due to the difficulty of conducting in-person surveys during the COVID-19 pandemic, we recruited participants through web portals and online communities, and a contactless survey was administered to adult volunteers who consented to participate in the study using a Google Form.

The sample size was determined based on Coates [20], who suggested that the sample size should be at least four times the minimum number of items or 200 and higher. Thus, the minimum sample size was set to 200 in this study, and 230 participants were recruited in consideration of potential dropouts. Of the 230 questionnaires, four were excluded for careless responses or failure to sign the consent form, resulting in a total of 226 questionnaires included in the final analysis.

### 2.3. Study Instruments

We obtained permission to use the C19P-S from Arpaci et al. [16]. We used the Fear of COVID-19 Scale developed by Ahorsu et al. [10] and adapted by Seong et al. [16] to evaluate the criterion validity of the K-C19PS, with reference to previous reports that fear is a predictor of a specific phobia [21,22].

#### 2.3.1. The K-C19PS

The K-C19PS was adapted from the tool developed by Arpaci et al. [16] to measure phobia in the general population not diagnosed with a phobia, based on the DSM-5 diagnostic tool for specific phobias. The scale has 20 items with four factors: psychological, psychosomatic, economic, and social. Each item is rated on a five-point scale ranging from “strongly disagree” (1) to “strongly agree” (5), with a higher score indicating greater phobia. Cronbach’s α was 0.93 for the overall scale, 0.88 for the psychological subscale, 0.90 for the psychosomatic subscale, 0.89 for the economic subscale, and 0.85 for the social subscale at the time of development. In this study, Cronbach’s α for the overall scale was 0.93.

#### 2.3.2. Fear of COVID-19 Scale

The Fear of COVID-19 Scale was originally developed by Ahorsu et al. [10] to measure the fear of COVID-19 in the general population and was adapted into Korean by Seong et al. [19]. The scale consists of seven items with two factors (physiological response to COVID-19 and emotional response to COVID-19). Each item is rated on a five-point scale from “never” (1) to “always” (5), and a higher score indicates greater fear of COVID-19. Cronbach’s α was 0.82 at the time of development, whereas Seong et al. [19] reported values of 0.87 for the overall scale, 0.86 for the physiological subscale, and 0.81 for the emotional subscale. Cronbach’s α was 0.86 for the overall scale in this study.

### 2.4. Study Procedure

#### 2.4.1. Cultural Adaptation of the Scale

The K-C19PS was adapted from the self-report C19P-S developed by Arpaci [16] to measure the psychological characteristics of individuals not diagnosed with a phobia, based on the DSM-5 diagnostic criteria for specific phobia.

The instrument was validated through a process involving translation, expert review, back-translation, pilot tests, interviews, and finalization. The translation was independently performed by the researcher and three Korean-native nurses and nursing professors who were fluent in English and Korean. During the expert review, the experts reviewed the items translated by three translators and reached an agreement on the final translations to develop the first draft of the Korean items.

The back-translation was performed by an English literature professor fluent in English and Korean. The professor had no prior knowledge of the original tool, which prevented bias and led to unexpectedly meaningful interpretations [23].

Subsequently, an English-native American compared the original tool with the back-translated tool to determine the congruence of meaning between the two. For incongruent items, the original translators reviewed and modified the Korean items, translated the items, and compared the back-translated English scale with the original scale.

A pilot test was conducted on 30 adults from December 3 to December 4, 2020, using the completed draft questionnaire. The participants were instructed to complete the self-report questionnaire, and the time required to complete the questionnaire was measured. After the pilot test, the participants were individually interviewed, and two students were found to have had difficulty answering item 2, “I understand my own emotions well,” and item 3, “I really understand what I feel.” One nursing expert and one English literature professor discussed the items and found that there were no problems in the translation and back-translation processes, as the participants were able to respond to the questions after reading the items twice. The Korean scale was finalized to 20 items without deleting these two items.

#### 2.4.2. Validity and Reliability Testing

Factorial (construct), convergent, and discriminant validities were investigated. Factorial validity was assessed using data from 226 participants. Exploratory factor analysis (EFA) was performed to examine the factors underlying COVID-19 phobia, agreement of these factors with those of the original scale, and the total variance explained by the construct. Subsequently, the results of the EFA were used to create a measurement model, and confirmatory factor analysis (CFA) was performed to examine whether the construct measured by each item was appropriate for measuring COVID-19 phobia.

Convergent validity was evaluated through correlation analysis based on the hypothesis that phobia and fear are significantly positively correlated. Item convergent validity was tested by confirming that average variance extracted (AVE) was greater than 0.05, and item discriminant validity was established based on whether the AVE of each factor was greater than the square of the correlation coefficient between the factors [24].

The reliability of the scale was evaluated using the internal consistency index Cronbach’s α and test-retest reliability by intraclass correlation coefficient (ICC).

### 2.5. Ethical Considerations

This study was approved by the Institutional Review Board at C University (CSIRB-20200014). The participants were adults aged 20 years or older who were recruited via a web portal and online communities because of the difficulty of conducting in-person surveys during the COVID-19 pandemic. The survey was administered using Google Forms after the participants signed an informed consent form. The participants were adequately informed about the purpose and content of the study, the freedom to withdraw from the study at any time without any disadvantages, and confidentiality and a guarantee of anonymity. After completing the survey, the participants were given a small gift.

### 2.6. Data Analysis

The study data were analyzed using SPSS/WIN 22.0 and AMOS 20.0. Participants’ general characteristics were analyzed using frequency, percentage, mean, and standard deviation, and the differences in the general characteristics between the participants in the EFA and those in the CFA were analyzed using a t-test or chi-square test. The validity of the tool was analyzed using factorial and convergent validities, and the items’ convergent and discriminant validities were evaluated.

The factors of the scale, items in each factor, item factor loadings, and explanatory power were examined through EFA. Factors were extracted via principal axis factoring, which is an appropriate method for extracting common factors because only common variance is used [25]. Factor rotation was performed to identify the specific factor with which each item was strongly associated; since there were correlations among the factors in this scale, the oblimin method of oblique factor rotation was used [25]. The suitability of the collected data for factor analysis was determined using the Kaiser–Meyer–Olkin (KMO) and Bartlett’s test of sphericity. The EFA was performed using data from 226 participants.

The CFA was performed to determine the fit of the model created based on the results of EFA, and all items were confirmed to have no missing values. The model fit was assessed using the χ^2^ (*p*-value), normed χ^2^ (Chi-square minimum/degree of freedom (CMIN/DF)), goodness of fit index (GFI), root mean square error of approximation (RMSEA), Tucker–Lewis index (TLI), and comparative fit index (CFI), which were used in the original scale’s development. CFA was performed using data from the 226 participants.

The convergent validity of the scale was assessed by analyzing Pearson’s correlation between the Fear of COVID-19 Scale, which was assessed for fear correlated with fear. Convergence and discriminant validity of the items were tested using AVE and compound reliability (CR) and the square of the coefficient of correlation between the factors.

Regarding the reliability of the scale, homogeneity was evaluated based on internal consistency using Cronbach’s α, and stability was evaluated using test-retest reliability and computing ICC.

## 3. Results

### 3.1. Participants’ General Characteristics

A total of 226 participants were enrolled in this study. 81 participants (35.8%) were men and 145 (64.2%) were women. The most common age group was 20–29 years (n = 100, 44.2%), followed by 30–39 years (n = 60, 26.5%), 40–49 years (n = 30, 13.3%), and ≥ 60 years (n = 8, 3.5%). The most common education level was high school graduation (n = 88, 38.9%), and there was one middle school graduate (0.4%), 24 college graduates (10.6%), and 24 graduate school degrees (10.6%). A total of 122 (54%) participants were single, and 103 (45.6%) were married. Regarding religion, 123 (54.4%) followed no religion, while there were 54 Protestant Christians (23.9%), 30 Buddhists (13.3%), 10 “other” (4.4%), and 9 Catholics (4.0%). The most common occupation was “other” (n = 110, 48.7%), followed by a profession (n = 39, 17.3%), public service (n = 26, 11.5%), and service (n = 10, 4.4%). Regarding perceived economic status, 127 (56.2%) reported medium, followed by medium-low (n = 55, 24.3%), medium-high (n = 55, 24.3%), low (n = 18, 8.0%), and high (n = 1, 0.4%). Regarding perceived health status, 138 (61.1%) claimed to have moderate health, 63 (27.9%) had good health, 20 (8.8%) had poor health, 4 (1.8%) had very good health, and 1 (0.4%) had very poor health. 27 participants had a chronic disease (11.9%), while 199 (88.1%) did not (Table 1).

### 3.2. Validity and Reliability Testing

#### 3.2.1. Content Validity

The content validity of the K-C19PS was evaluated by two psychiatric nursing professors and three clinical practitioners. The content validity index was 1.0, based on which all 20 items were included in the final questionnaire.

#### 3.2.2. Construct Validity

**Figure 1:**
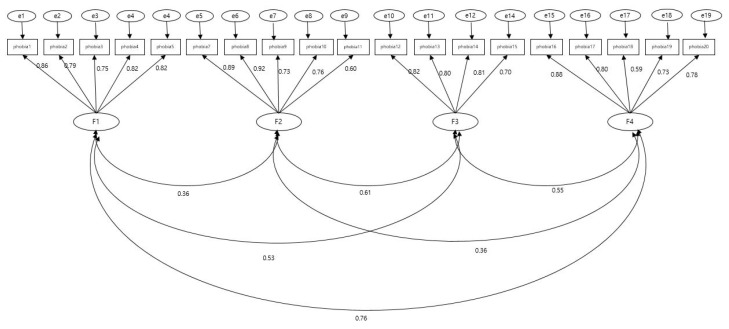
Confirmatory factor analysis (CFA).

The construct validity of the K-C19PS was tested by performing CFA with the four factors proposed in the original tool to create Model 1. Standardized factor loadings of all 20 items were below 0.50, and one item (item 6) that diminished the overall model fit was deleted to create Model 2. The standardized factor loadings of 19 items in the modified model 2 ranged from 0.59 to 0.89 (Figure 1).

The normality of the data was tested before performing the CFA using Model 2. The skewness ranged from −1.17 to 1.88, while kurtosis ranged from −0.02 to 5.05, which met the criteria of an absolute value of skewness of <3 and an absolute value of kurtosis of <10, thereby satisfying the assumption of univariate normality [26].

In the multivariate normality test, multivariate kurtosis was 128.94, with a threshold of 34.31, which exceeded the cutoff of 5.99, thereby failing to meet the assumption of multivariate normality [26]. Thus, the parametric estimation and the effect analysis were performed with bootstrapping to analyze data violating the assumption of multivariate normality. The significance of the model paths was analyzed using regression coefficients and *p*-values [27]. The chi-square value was 440.93 (df = 146, *p* < 0.001). A normed chi-square of 3 or lower is considered acceptable [25]; our value of 3.02 was thus acceptable.

Regarding the model fit indices, the absolute fit indices were RMSEA = 0.10, GFI = 0.83, RMR = 0.07, and SRMR = 0.07, and incremental fit indices were: CFI = 0.90, IFI = 0.90, NFI = 0.85, and TLI = 0.88, which did not meet the criteria for acceptable fit. Thus, Model 3 was created after establishing correlations between measurement errors for exogenous latent variables. The fit of Model 3 was as follows: Chi-square = 265.67 (df = 138, *p* < 0.001), while normed chi-square decreased to 1.93, thereby meeting the criterion for acceptability of a value of 3 or below, with a value of 2 or below denoting good fit [25]. The fit indices were RMSEA = 0.06, GFI = 0.89, RMR = 0.05, and SRMR = 0.06, with incremental fit indices of CFI = 0.96, IFI = 0.96, NFI = 0.91, and TLI = 0.95, indicating good fit for all items. Thus, the construct validity of the K-C19PS, consisting of 19 items with four factors, was established (Table 2).

#### 3.2.3. Convergent Validity

The convergent validity of the K-C19PS was evaluated using the standardized factor loading, AVE, and CR (Table 3). The standardized factor loadings of 19 items ranged from 0.59 to 0.89, meeting the cutoff of ≥ 0.50 for all items. The CR ranged from 0.83 to 0.91, and the AVE ranged from 0.55 to 0.66. Thus, the convergent validity of the items was established [27].

#### 3.2.4. Discriminant Validity

The discriminant validity of the K-C19PS was evaluated by comparing the AVE of the two constructs and the square of the correlation coefficient between two constructs, where discriminant validity is deemed established if AVE is greater than the square of the correlation coefficient [25]. The results confirmed that the square of the correlation coefficients between factors was smaller than the AVE of each factor, and thus the discriminant validity was established (Table 4).

#### 3.2.5. Reliability

The internal consistency of 19 items in the K-C19PS was examined; Cronbach’s α for the 19 items was 0.93. By subscale, the Cronbach’s α was 0.90 for the psychological subscale, 0.87 for the psychosomatic subscale, 0.86 for the economic subscale, and 0.87 for the social subscale (Table 4).

#### 3.2.6. Criterion Validity

To test the criterion validity of the K-C19PS, we analyzed the correlation between the K-C19PS and the Fear of COVID-19 Scale. The results confirmed that there was a statistically significant positive correlation (r = 0.80, *p* < 0.001). As a correlation coefficient between 0.40 and 0.80 is considered to confirm the criterion validity [28], the criterion validity of the K-C19PS was established.

## 4. Discussion

This study translated the C19P-S, a scale developed to measure COVID-19 phobia, into Korean and presented primary evidence for the reliability and validity of the scale by evaluating its content validity, item analysis, construct validity, convergent validity, discriminant validity, criterion validity, and reliability.

Four factors were confirmed through the CFA. The original tool consisted of psychological, psychosomatic, economic, and social subscales, and these four factors were also confirmed for the K-C19PS. This suggests that the items in the translated tool convey the same meaning and describe the factors as those in the original tool and that each item delivered accurate meaning following the translation process.

We created a model for latent variables and items and analyzed the fit of the model. The model had a chi-square value of 265.67 (df = 138, *p* < 0.001), normed chi-square of 1.93, RMSEA of 0.06, and GFI of 0.89, with incremental fit indices of CFI = 0.96, IFI = 0.96, NFI = 0.91, and TLI = 0.95, confirming that all indices indicated a good fit. The original tool had a chi-square value of 446.93 (df = 125, *p* < 0.001), normed chi-square of 3.575, RMSEA of 0.04, and GFI of 0.98, with incremental fit indices of CFI = 0.99, IFI = 0.99, NFI = 0.98, and TLI = 0.98. Based on the criteria that a normed chi-square of 3 or below indicates an acceptable fit and a value of 2 or below indicates a good fit [25], the K-C19PS had a better fit than the original tool.

In this study, item 6, “I passionately argue with (talk to) people who act irresponsibly despite the COVID-19 pandemic,” which had the lowest factor loading, was deleted. This may be because of the cultural tendency of Koreans toward fostering an unconditionally accepting and trusting relationship with the people within their academic, hometown, and family networks wherein they freely express their opinions; however, they tend to adopt an attitude of “I will see what you do” based on mutual mistrust toward distant acquaintances or strangers [29]. Further, item 16, “Since the COVID-19 outbreak, I become extremely anxious when I see people cough,” had a high factor loading in this study. This may be attributable to the altered health and hygiene environment and public awareness recognizing the importance of face masks. This suggests that campaigns to promote proper coughing etiquette were effective.

The convergent and discriminant validities of the items were tested using standardized factor loading, CR, and AVE. The results indicated that the items consistently measured the construct of interest, and the factors were mutually independent. However, in the original tool, factors 1 and 4 were strongly correlated (r = 0.71), which differed from our study (r = 0.56). This may be a result of deleting item 6, which had the lowest factor loading. The deletion of item 6 ensured independence among the factors. That is, we confirmed that the five items of the psychological subscale, five items of the psychosomatic subscale, four items of the economic subscale, and five items of the social subscale were appropriate for measuring their corresponding factors and were distinct from the items included in other factors. Therefore, the construct validity of the K-C19PS with 19 items and four factors was considered established.

The baseline validity of K-C19PS was assessed by analyzing its correlation with fear on the COVID-19 scale. Typically, 0.40 to 0.80 is recommended [27], and a low correlation coefficient should be interpreted with care as the tool may measure phenomena different from those measured by external criteria [29]. There was a statistically significant positive correlation between the two tools (r = 0.80, *p* < 0.001), suggesting that the baseline validity of K-C19PS was established.

The reliability of the tool was analyzed based on its internal consistency. Cronbach’s α was above 0.80 for all subscales, with 0.90 for the psychological subscale, 0.87 for the psychosomatic subscale, 0.86 for the economic subscale, 0.87 for the social subscale, and 0.93 for the overall scale. The original tool had a Cronbach’s α value of 0.88 for the psychological subscale, 0.90 for the psychosomatic subscale, 0.88 for the economic subscale, 0.85 for the social subscale, and 0.93 for the overall scale. A similar level of Cronbach’s α between the two scales shows that the K-C19PS has high internal consistency.

This study could encourage active research on the psychosocial aspects of COVID-19 response, which have been greatly neglected in various fields, including nursing, by developing K-C19PS and validating tools with Korean adults. In addition, K-C19PS takes about 5–10 min to complete the questionnaire with 19 items, covering all aspects of psychological, mental, physical, economic and social aspects. However, the traditional Fear of COVID-19 Scale is made up of nine items made up of one element, so the K-C19PS can be more useful as it can comprehensively measure its composition across different aspects.

The strength of this study is that it was the first to validate K-C19PS. We expect this scale to be useful in research and nursing to assess the psychosocial impact of COVID-19. Furthermore, if this tool can be used to accurately measure the level of COVID-19 phobia of adults and provide appropriate intervention programs for them, it will be possible to prevent psychosocial problems caused by fear and contribute to health promotion. In addition, it is expected that it will be able to function sufficiently as a measurement tool to provide an intervention program to reduce the phobia of pandemics and to see the difference in its effect.

## 5. Conclusions

In this study, the K-C19PS was developed by translating and back-translating the C19P-S, and the content validity, criterion validity, convergent and discriminant validity, construct validity, and reliability of the K-C19PS were analyzed. The scale was finalized to 19 items, and the reliability and validity of the scale were confirmed. Cronbach’s α was above 0.80 for all subscales, with 0.90 for the psychological subscale, 0.87 for the psychosomatic subscale, 0.86 for the economic subscale, and 0.87 for the social subscale, and reliability was confirmed based on internal consistency and split-half reliability. Satisfactory criterion validity was established for the K-C19PS based on its correlation with the Fear of COVID-19 Scale. We anticipate that the K-C19PS will be actively utilized in subsequent studies in the fields of healthcare and nursing. This study is meaningful in its verification of the reliability and validity of the Korean translation of Arpaci et al.’s [16] tool, which can measure fear of COVID-19 in the Korean culture; the tool may be of use to Korean nursing science. In addition, the tool reflects psychological, psychosomatic, economic, and social aspects of fear of COVID-19, including components not considered in existing fear measurement tools. Therefore, the tool developed in this study better reflects the specific nature of the current situation and can be effectively used to assess people’s fears in the context of COVID-19 in Korea.

## 6. Limitations

This study has limitations in that it did not assess cross validity for the same subjects in the exploratory and confirmatory factor analyses. Therefore, future research should continue to refine, validate, and assess the reliability of the tool. In addition, although this study considered adults in the general population, to increase the accuracy and applicability of the tool, development should be repeated for specific groups, such as those with specific diseases.

Based on the findings, we suggest the following: First, subsequent studies should analyze the relationship between various psychosocial parameters and COVID-19 phobia. Second, subsequent studies should develop and assess the effectiveness of nursing intervention programs for anxiety management, considering the factors identified in this study.

## Figures and Tables

**Table 1:** General Characteristics (*N* = 226).

Characteristics	Categories	*n*(%)
Gender	Male	81(35.8)
	Female	145(64.2)
Age(group)	Twenteen	100(44.2)
	Trigenerian	60(26.5)
	Forty~Fifty	30(13.3)
	Sixty and older	8(3.5)
Education level	Middle school	1(0.4)
	High school	88(38.9)
	University	113(50.0)
	Graduation school	24(10.6)
Married	Single	122(54.0)
	Married	103(45.6)
	The others	1(0.4)
Religion	Protestantism	54(23.9)
	Catholic	9(4.0)
	Catholic	30(13.3)
	None	123(54.4)
	The others	10(4.4)
Job	Profession	39(17.3)
	Service	10(4.4)
	Official	26(11.5)
	Businessman	41(18.1)
	The others	110(48.7)
Economic state	Advanced	1(0.4)
	Upper-intermediate	25(11.1)
	Intermediate	127(56.2)
	Low-intermediate	55(24.3)
	Low	18(8.0)
Health state	Very bad	1(0.4)
	Bad	20(8.8)
	Normal	138(61.1)
	Good	63(27.9)
	Very good	4(1.8)
Presence of chronic disease	Yes	27(11.9)
	No	199(88.1)

**Table 2:** Analysis of Construction Validity.

	χ^2^(*p*)	df	CIMIN/df	RMSEA	RMR	SRMR	GFI	IFI	TLI	CFI
Model 1	459.88(*p* < 0.001)	164	2.80	0.90	0.07	0.09	0.83	0.90	0.88	0.90
Model 2	440.93(*p* < 0.001)	146	3.02	0.10	0.07	0.07	0.83	0.90	0.88	0.90
Model 3	265.67(*p* < 0.001)	138	1.93	0.06	0.05	0.06	0.89	0.96	0.95	0.96

**Table 3:** Analysis of Convergent Validity of Items (*N* = 226).

Factors	Items	Standardized Estimates	Nonstandardized Estimate	SE	CR	*p*	AVE	Construct Reliability
Psychological	Item1	0.86	1.00	-	-	-	0.66	0.91
	Item2	0.79	0.99	0.07	14.35	<0.001		
	Item3	0.76	1.01	0.08	13.44	<0.001		
	Item4	0.82	1.10	0.07	15.37	<0.001		
	Item5	0.82	1.03	0.07	15.25	<0.001		
Psyhosomatic	Item7	0.89	1.00	-	-	-	0.64	0.89
	Item8	0.92	0.96	0.05	19.87	<0.001		
	Item9	0.73	1.10	0.08	13.38	<0.001		
	Item10	0.76	0.86	0.06	14.18	<0.001		
	Item11	0.60	1.01	0.10	10.09	<0.001		
Economic	Item12	0.82	1.00	-	-	-	0.62	0.87
	Item13	0.80	1.08	0.08	12.98	<0.001		
	Item14	0.81	0.87	0.07	13.17	<0.001		
	Item15	0.70	0.86	0.08	11.03	<0.001		
Social	Item16	0.88	1.00	-	-	-	0.55	0.83
	Item17	0.81	0.87	0.06	14.84	<0.001		
	Item18	0.59	0.50	0.05	9.60	<0.001		
	Item19	0.73	0.82	0.06	12.83	<0.001		
	Item20	0.78	0.76	0.05	14.12	<0.001		

**Table 4:** Correlation Matrix and Reliability of K-C19P Scales.

Variables	Factor1	Factor2	Factor3	Factor4
	r(*p*)	r(*p*)	r(*p*)	r(*p*)
Factor1	**0.66**			
Factor2	0.20	**0.64**		
Factor3	0.32	0.48	**0.62**	
Factor4	0.56	0.19	0.34	**0.55**
Cronbach’s α	0.90	0.87	0.86	0.87
Cronbach’s α(Total)	0.93			

Bold: different from other data.

## Data Availability

No new data were created or analyzed in this study. Data sharing is not applicable to this article.
